# Supplementary material for: Retinal curl as a functional signal for heading estimation beyond the focus of expansion
Source: eLife. 2026 Sep 25;15:RP110770. doi: 10.7554/eLife.110770 (PMC13614786; doi:10.7554/eLife.110770)
Supplement: Peer review history

# Editor report: eLife Assessment

**DOI:** 10.7554/eLife.110770.3.sa0

**Authors:** Marisa Carrasco

**Affiliations:** New York University United States

**Keywords:** Convincing, Important

This study provides an **important** and biologically plausible account of how human perceptual judgments of heading direction are influenced by a specific pattern of motion in optic flow fields known as retinal curl. By combining psychophysical experiments and neural modeling, the authors demonstrate that what was previously considered an incidental, 'nuisance' signal actually serves as a functional control signal for estimating heading and steering toward a fixated target. The evidence supporting the role of curl signals is **convincing** and advances our understanding of vision-based navigation at the behavioral level. A particular strength of the work is the direct manipulation of curl within flow fields, demonstrating that it effectively cancels or reverses heading biases. This work provides an invaluable framework for future exploration into the neural mechanisms of steering control based on retinal curl.

# Referee report: Reviewer #1 (Public review)

**DOI:** 10.7554/eLife.110770.3.sa1

Summary:

This carefully executed study uncovers the functional relevance of curl signals that impinge on the retina every time an observer's gaze direction and movement direction are not aligned. This finding is important, highlighting the functional role of an abundant incidental signal (curl in retinal motion) that has thus far believed to be a nuisance that needs to be filtered out of the retinal motion stream. As such, the study forms an important contribution to the emerging recognition that incidental sensory signals are not a challenge to the sensorimotor system, but contain functionally relevant and effectively used visual signals. The study's evidence is compelling: A combination of psychophysical experiments and critical manipulations, control theory and neural modeling makes an internally consistent and biologically plausible case for the role of curl signals in estimating heading direction. The experimental and modeling results clearly go beyond previous studies and significantly advance our understanding of vision-based navigation.

Strengths:

The study has its strengths in the combination of psychophysical experiments and critical manipulations, control theory and neural modeling, which together make an internally consistent and biologically plausible case for the role of curl signals in estimating heading direction.

This study uncovers the functional relevance of curl signals that occur on the retina when an observer is moving and gaze is not straight ahead. The experimental and modeling results clearly go beyond previous studies and significantly advance our understanding of vision-based navigation.

Another clear strength is that the study uses tightly controlled experimental manipulation to provide strong test cases for the hypothesis that curl is used for visual navigation. These conditions are important to constrain the proposed model (and future models) of heading control.

The modeling is very clearly described and the modeling and analysis code is published and freely available. The authors go beyond a back-of-the-envelope control model and show how it might be implemented at the neural-circuit level. The model is biologically plausible.

Weaknesses:

I see no major weaknesses of the study. I expect it to inspire future research that extends these findings to a wider range of visual environments (including walking in natural scenes), motion speeds and kinds of movements.

Comments on revised version.

I have no additional comments for the authors.

# Referee report: Reviewer #2 (Public review)

**DOI:** 10.7554/eLife.110770.3.sa2

This study examines how curl in the retinal flow field can be used as a control variable for estimating and controlling the heading of a moving observer. The basic idea (which is not entirely new, see Matthis et al. 2022) is that translation along a path with eccentric gaze (meaning that the subject is not heading toward the point they are looking at) produces a pattern of optic flow on the retina with a rotational component around the point of fixation (which can be captured by the mathematical "curl" operator). The sign and magnitude of retinal curl varies with heading relative to the point of fixation, such that curl can be used as a control variable to steer rightward or leftward to move toward the fixated target. The authors perform behavioral experiments and show that there are biases in perceived heading that seem to be largely governed by retinal curl. They also show that a simple controller model can use curl to steer toward a target, and they provide a neural network model that provides a biologically-plausible implementation of the controller (although there are some questions about that).

There is a core of interesting work here that I think can be important to the field. However, there is a lack of clarity on several important fronts, including design of the behavioral experiments, presentation of the behavioral data, conceptual framing of what curl can and cannot do, etc. Equally importantly, the manuscript is not written in a manner that will make it accessible to most vision scientists. I consider myself to be pretty knowledgeable about optic flow, and I had to read most of the manuscript 3 or 4 times to be able to understand the bulk of it. And my experience is that most vision scientists do not understand optic flow well, so I fear that most of the readers that the authors should want to reach would struggle to understand the work. As written, this is mainly going to make an impact on a handful of optic flow gurus. Thus, this manuscript is going to need a major overhaul to clarify important issues and make this more accessible.

Major issues:

(1) The manuscript contains inconsistent, if not misleading, messaging about what information retinal curl does, and does not, provide regarding heading estimation. In the Abstract, the authors state: "We propose an alternative: the visual system utilizes retinal curl directly to estimate heading, rendering the explicit recovery of the FOE unnecessary." Based on my understanding of the rest of the manuscript, I find this statement to be a misrepresentation for two main reasons:

a. To "directly estimate heading" relative to what? When not qualified, most people interpret "heading" to mean an observer's heading relative to the world (or some allocentric reference frame). But retinal curl only gives information about an observer's heading relative to the point on which their eyes are fixated. Moreover, that point of fixation will change every few hundred milliseconds in natural viewing, so the retinal curl will change with each new fixation even as heading relative to the world remains unchanged. So, I think most readers would grossly misinterpret the claim that retinal curl can be used "directly to estimate heading". Indeed, in the authors' controller model, the initial heading needs to be given and then the controller can work. But from where does the visual system get the initial heading, since it does not come from curl? These issues are left hanging. Thus, while curl can provide a very useful input for steering toward a fixated target, other signals are needed to estimate heading relative to the world. This has to be made much clearer early on, and a conceptual schematic diagram might help. Also, the authors generally do not specify the reference frame of the variables they are talking about, leaving lots of room for misinterpretations. It should be clear each time they are talking about a variable, such as heading, whether it is relative to the fixation target, body, world, etc.

b. It seems to me that retinal curl will depend on other variables, in addition to heading relative to the fixation target. For example, it seems to me that the magnitude of retinal curl will depend on self-motion speed, the depth structure of the scene, the angle of elevation of the fixated target, and perhaps others. This is not discussed at all, and many readers would get the misguided impression that there is a 1:1 mapping from curl to heading (relative to fixation). If I am right that this is not correct, it means that retinal curl can tell the observer whether to steer right or left to move toward the fixated target, but it cannot tell them how much to steer. Indeed, in the authors' controller model, there is a free parameter that calibrates curl to angle. It makes sense that this works to fit trajectory data that are given from a fixed environment, but it is unclear how the brain would use retinal curl to control steering when these other variables are uncertain or changing unpredictably. Moreover, how does the system change the mapping from curl to steering command as the location of fixation changes relative to the current heading? These are issues that need to be brought up in framing the problem and discussed at some length. If the authors can show mathematically that retinal curl is only dependent on heading (relative to fixation) and not any of these other variables, it would be very valuable to show the equations for this relationship.

(2) The description of the behavioral experiment and presentation of behavioral data leaves a lot to be desired.

a. First, it is stated (line 158) that "Participants continuously reported their perceived direction of self-motion while maintaining fixation on the yellow dot." Again, reference frame is completely unspecified. Participants were reporting their perceived heading relative to what? The fixation target? The world? What exactly were the instructions given to the subjects to perform the task? Based on the description of how perceived paths are computed (line 166-), it seems to be presumed that subjects are reporting their heading relative to the world because those angles are then converted into x and z coordinates in what I presume is a world-centered reference frame. But how do we know that subjects are accurately reporting their heading relative to the world? What if they are biased in their reports by the location of the fixation target relative to the scene, or by some other reference signal? Is it possible for the authors to rule out the possibility that perceptual biases seen in the unaltered curl condition result from observers not fully adopting the assumed reference frame of the task? If this cannot be firmly excluded, it seems to create problems for the rest of the study.

b. I also feel that there is a mismatch between what the behavioral task requires and what the controller model does. Subjects are apparently asked to report their heading relative to the world, but the controller model only controls their heading relative to the point that they are fixating. I understand how this is resolved in the model, but I think this type of distinction is buried and will not be apparent to most readers. Again, the reference frames of what is being measured and controlled need to be specified explicitly in all parts of the paper, and the authors needs to explain how the system would combine curl-based control with some other measures of (at least initial) heading for world-centered heading to be computed. All of the assumptions need to be clearly specified.

c. Second, I found it frustrating that the authors never present raw perceptual data from the observers. Rather, in Figure 2, we see reconstructed trajectories that are perfectly smooth with no indications of noise whatsoever. Since these paths are computed from the perceptual reports, there must be some noise inherent in them. The figures should represent this uncertainty somehow, and it should be explained how these perfectly smooth trajectories are obtained.

(3) "...the magnitude of retinal curl in the fovea can specify the body trajectory relative to gaze (Matthis et al., 2022)." The main idea put forward by the authors here seems to overlap heavily with this statement that they attribute to Matthis et al. 2022. While I think this paper still adds importantly to the topic, the authors do not discuss how their findings are different from those of Matthis et al. 2022, why they are an important extension, etc. Readers should not have to go read this other paper to have any idea how the present findings are placed in importance relative to the literature.

(4) The analysis and treatment of eye movements is extremely weak. The authors discarded trials for which gaze deviated from the fixation point by more than 3 degrees (which is a LOT given that the eye speeds are generally in the neighborhood of 0.5 deg/sec), and they provide basic stats on the distribution of positions. But this largely misses the point: it is not small position errors that are likely to matter, but rather velocity errors. Even a small amount of retinal slip of the target while it is being pursued will cause image motion that is going to alter the optic flow field around the fixation target. So, for example, the retinal curl field may no longer be centered on the fixation target. How do we know that some of the perceptual biases are not influenced by image motion resulting from imperfect tracking of the fixation target? This needs to be analyzed and discussed.

(5) I found the sections of text comparing the separate and joined fits (starting line 287) to be a bit too rosy. The authors show the separate fits in the main text, and it is not very surprising that these fits are good given that the model has 30 parameters, and these data are pretty low dimensional. The authors only show the joined fits in the supplement, and they say that they are almost as good as the separate fits (indeed they are better in a model comparison sense, but this is 30 parameters vs. 2 parameters). However, when I look at the fits of the joined model in the supplement, I don't find them to be very impressive. In particular, the model grossly misses the data for the straight paths for several subjects (e.g., id5, id6, id8, id10). And fitting the straight paths would presumably be easiest. This implies that the joined model is really missing something and that fitting the curved paths interacts strongly with fitting the data for different fixation target locations on the straight path. I think that the authors should discuss the results a bit more soberly and tone down their conclusions here.

(6) The section of the paper on neural simulations (starting line 387) has a few weaknesses. First, why are only straight paths simulated here? This does not seem to provide a very rigorous test of the model. Second, it is awkward that the simulation results are presented in units of pixels, rather than degrees. Third, the authors seem to downplay the fact that the neural estimates of heading seem to oscillate rather wildly (over a range of hundreds of pixels, whatever that means, see especially Fig. S16). It was far from clear to me how an estimate of heading with these large oscillations is useful. It would seem to require that heading estimates are integrated over substantial lengths of time to be reliable. It was therefore unclear how the model produces such smooth paths from these oscillating estimates.

Comments on revised version.

Overall, the authors have done a responsible job of responding to the comments of my previous review, and the manuscript is substantially improved. There are a few points on which I still do not completely agree with the authors, and I think these are important to document for the record:

(1) Introduction: "Pure visual decomposition should function regardless of 3D depth or whether the rotation stems from an active eccentric fixation." Perhaps in a world of noiseless perfect computation, this might be true. But I generally disagree. When there is more depth structure in an environment, then translation of the observer is generally going to create greater motion parallax. That is a fact that I don't think can be disputed. And greater motion parallax should help to decompose optic flow into components related to translation and rotation (the latter of which is not depth dependent), especially when there is noise in estimating location motion vectors.

(2) Related to point #9 of my previous review: I had asked why the authors believed that retinal curl was computed in area MSTd. Their response is that previous studies (i.e., Graziano et al. 1994) show selectivity to spiral motion stimuli in MSTd. That is true, but those studies typically placed the spiral stimulus centered on the MSTd receptive field, hence they were not presenting something like retinal curl as defined here. So, I think it is still an open question as to where in the brain retinal curl is encoded, and from which areas it would be possible to decode retinal curl from population responses.

(3) Related to point #10 of my previous review: I had asked about biological plausibility of the gaze-centered inhibition signal in the model. The authors' response is that parietal neurons show gain fields in which response depends (usually monotonically) on eye position. This is true, but it is not a trivial jump from gain fields in individual neural responses to a gaze-centered inhibition signal, and I think the authors should have been more forthcoming about the lack of an established neural signal that directly signals what they want in their model.

(4) The authors point out that the perceptual biases they measure take a few seconds to emerge and they attribute this to temporal integration. But in their curl manipulations, they temporally average over a 2.4 second window in computing the curl signals that they use to cancel or over-cancel curl. So, it is not clear whether some of the delay in the behavioral effects might result from their computations.

(5) Related to point #13 of my previous review: I had asked about empirical evidence for the assumption of a relationship between the heading preferences of MSTd neurons and their receptive field locations. In response, the authors state that such a relationship is built into the Layton and Browning (2014) model. While that is a precedent, citing another model as a response to a question about empirical evidence is not a convincing response. If there is no empirical evidence to support such a relationship, it would have been better for the authors to acknowledge this.

Given the way that the eLife review model works, it is not necessary for the authors to address these comments, but I think they should be included in the public review record.

# Referee report: Reviewer #3 (Public review)

**DOI:** 10.7554/eLife.110770.3.sa3

Major strengths include the use of realistic retinal motion recorded during virtual walking, an elegant manipulation of curl, converging behavioral and modeling evidence, and grounding in control theory. This provides a novel and important contribution to our understanding of how the brain processes motion information and intuition about how that information might be used to guide steering. In addition, they provide a computational mechanism by which retinal flow curl can be used as a control signal.

The revised ms has been strengthened by more explicit discussion of the literature where there has been mixed evidence for the use of the Focus of Expansion. Since the ms is a strong test of the use of curl as a heading signal, this allows a deeper understanding of the importance of the finding and historical context. The ms has also been strengthened by a more explicit discussion of integration of the time-varying signal over periods of several seconds, which is an important demonstration. The implications of the ms are still a little unclear, as the results involve visual judgements in seated subjects. The use of different sources of information when humans walk from one place to another in real life may be complex and involve a variety of different sources of information.

# Author comment: Author response

**DOI:** 10.7554/eLife.110770.3.sa4

**Authors:** Kontessa I Zorpala, Joan López-Moliner

**Affiliations:** Universitat de Barcelona Barcelona Spain; Universitat de Barcelona Barcelona Spain

The following is the authors’ response to the current reviews.

We thank the editors for their positive assessment of our manuscript, and all the referees for their constructive comments, which have substantially improved this work. We welcome the opportunity to address referee #2's points for the public record, as they highlight key theoretical nuances and valuable future research directions.

(1) We appreciate the reviewer's point that, in a noisy biological system, the increased motion parallax provided by a rich 3D depth structure naturally aids in separating translation from rotation. We fully agree on this point. Our argument aimed at highlighting a fundamental theoretical distinction. Pure algebraic decomposition algorithms are mathematically capable of solving heading on flat planes. The fact that human perception often shows biases in these zero-depth conditions, unless extra-retinal cues are present, suggests that the visual system does not rely on a generalized, global de-rotation algorithm. Instead, it relies on heuristic, depth-dependent structural signals (like motion parallax and retinal curl). We maintain that while depth certainly reduces noise, its strict necessity points toward an ecologically grounded control strategy rather than a noisy global decomposition process.

(2) We think the reviewer raises a valid point regarding the exact neural locus of retinal curl encoding. It is true that Graziano et al. (1994) utilized centred spiral stimuli rather than the spatially offset curl geometries defined in our task. We view the spiral tuning of MSTd not as a direct, one-to-one mapping of full-field retinal curl, but rather as the foundational computational building block required to extract such a signal. We fully agree with the reviewer that identifying exactly where and how this population response is decoded into a unified, gaze-relative retinal curl signal remains an exciting and open empirical question for future neurophysiological research.

(3) We acknowledge the reviewer's call for transparency here. The transition from well-documented multiplicative gain fields (which modulate response amplitude based on eye position) to a direct, localized gaze-centered inhibitory drive is indeed a theoretical abstraction in our model. We utilized this localized inhibition as a functional mechanism to demonstrate how sensory evidence and spatial priors might competitively interact within a standard Mexican-hat recurrent architecture. While gain fields clearly establish that parietal networks integrate gaze position, we agree that the exact local-circuit implementation mapping these gain fields to the specific inhibitory dynamics we modeled has yet to be empirically established.

(4) The reviewer smartly questions whether the 3-5 second behavioral biases delay emerges from the 2.4-second smoothing window used in our computational flow manipulation. It is important to clarify that this 2.4-second window was used solely to stabilize the computed curl signal against high-frequency gait oscillations. This smoothing was restricted strictly to the modeling phase of the controller and neural model and was not applied to the participants' responses. The gradual build-up of their perceptual bias over 3-5 seconds represents their own intrinsic temporal integration of this trajectory, independent of the smoothing parameters used to smooth the curl used in the fitting of the controller and neural modelling.

(5) We concede the reviewer's point that citing a computational model (Layton & Browning, 2014) does not constitute direct empirical evidence for a relationship between MSTd heading preferences and their receptive field locations. Our intention was to highlight a successful theoretical framework that elegantly organizes known properties of MSTd into a system capable of bypassing global de-rotation. We readily acknowledge that direct, single-cell empirical validation of this specific topographic relationship is currently lacking in the literature, and we appreciate the reviewer ensuring this distinction is clearly noted for the record.

The following is the authors’ response to the original reviews.

**eLife Assessment**

This study provides an important and biologically plausible account of how human perceptual judgments of heading direction are influenced by a specific pattern of motion in optic flow fields known as retinal curl. By combining psychophysical experiments and neural modeling, the authors demonstrate that what was previously considered an incidental "nuisance" signal actually serves as a functional control signal for estimating heading and steering toward a fixated target. While the evidence for the role of curl signals is convincing and advances our understanding of vision-based navigation, the work's impact would be strengthened by situating these findings among other cues that contribute to heading estimation, and by clarifying both the time course of these computations and their generalizability across different navigational contexts.

We thank the editors and reviewers for their insightful feedback and positive assessment of our study. In this revised version, we have made substantial modifications to better situate our findings within the broader landscape of cues contributing to heading estimation, while also clarifying the time course of these computations and their generalizability across different navigational contexts. These points are included in new sections in the revised discussion**.**

In addition, and following eLife guidelines, we have moved the methods to the end and make sure that the manuscript reads well without needing to go through methods first.

Next, we address all the concerns raised by the reviewers.

**Reviewer #1 (Public review):**

We appreciate Reviewer #1’s very positive feedback. Incorporating the perspective of ‘incidental’ sensory signals is a valuable suggestion that aligns perfectly with our findings. We agree that this perspective significantly strengthens the impact of our paper.

In the revised version we have added a last section in the Discussion (Generalizability and Testable Predictions) to comment on the functional utility of 'incidental' signals and incorporated the suggested references. In addition, in the same heading, we briefly elaborate on the predictions and generalizability of the model and possible manipulations that might affect the integration between sensory evidence (curl signal) and straight-ahead prior.

**Reviewer #1 (Recommendations for the authors):**

It would be great if the authors could discuss the implications and predictions of their model.

First, from a broader perspective, the study forms an important piece in the emerging recognition that incidental sensory signals are not a nuisance to the sensorimotor system, but contain functionally relevant and effectively used visual signals (Rolfs & Schweitzer, 2022). The authors may want to appreciate their contribution to this perspective in the discussion of the impact of their results. Indeed, a similar shift in perspective has been realized in the recognition that saccade-induced motion signals are not entirely suppressed but play a functional role for gaze correction (Schweitzer & Rolfs, 2021).

Second, the authors could spell out additional predictions of their proposal: What are experimental manipulations that could shift the balance between relying on a straight-ahead prior and sensory estimation of curl? What would happen in extreme cases of such sensory evidence? When would priors become overwhelmingly influential?

As commented in the public review we have now included these two aspects in the discussion.

Minor point: After equation 11, the authors may want to specify that, like position, gaze g is also coded as {x,y}, just like position p.

While the neural model details have been moved to Appendix 2 (including this equation), we added text before Equation 22 (previously Eq. 11) clarifying that gaze is encoded in image coordinates. We omitted point index *i* because the equation applies to all image points relative to a given gaze g.

**Reviewer #2 (Public review):**

We appreciate the reviewer’s feedback regarding the formalization of our reference frames. We agree that certain definitions were implicitly assumed rather than explicitly stated. We have revised the manuscript to provide all necessary self-contained information, ensuring that the geometry of the task response and the definition of heading are unambiguous. In the last paragraph of the revised introduction, we make clear the response frame of reference which is also included in the caption of fig 1. Also, we have addressed the gap between the task response (in world coordinates) and the functional role of the controller. This is particularly discussed in the discussion (section: reference frames) in which we also provide (and rule out) potential alternatives to our response biases. We also address all the other points raised by the reviewer.

Major issues:

(1) The manuscript contains inconsistent, if not misleading, messaging about what information retinal curl does, and does not, provide regarding heading estimation. In the Abstract, the authors state: "We propose an alternative: the visual system utilizes retinal curl directly to estimate heading, rendering the explicit recovery of the FOE unnecessary." Based on my understanding of the rest of the manuscript, I find this statement to be a misrepresentation for two main reasons:

(a) To "directly estimate heading" relative to what? When not qualified, most people interpret "heading" to mean an observer's heading relative to the world (or some allocentric reference frame). But retinal curl only gives information about an observer's heading relative to the point on which their eyes are fixated. Moreover, that point of fixation will change every few hundred milliseconds in natural viewing, so the retinal curl will change with each new fixation even as heading relative to the world remains unchanged. So I think most readers would grossly misinterpret the claim that retinal curl can be used "directly to estimate heading". Indeed, in the authors' controller model, the initial heading needs to be given, and then the controller can work. But from where does the visual system get the initial heading, since it does not come from curl? These issues are left hanging. Thus, while curl can provide a very useful input for steering toward a fixated target, other signals are needed to estimate heading relative to the world. This has to be made much clearer early on, and a conceptual schematic diagram might help. Also, the authors generally do not specify the reference frame of the variables they are talking about, leaving lots of room for misinterpretations. It should be clear each time they are talking about a variable, such as heading, whether it is relative to the fixation target, body, world, etc.

In our study, participants were instructed to report their “perceived direction of self-motion” by aligning a rotational encoder (steering wheel) with the direction they felt they were moving within the 3D simulated scene. Consequently, participants reported their instantaneous heading in a world-centered reference frame, from which the 3D trajectories were reconstructed. Since the reviewer had to infer this information, we have clarified this point at the end of the introduction, legend of figure 1, methods (now at the end of the ms.) and discussion to ensure it is immediately evident.

Participants were informed that the initial heading (i.e. θ_0_ in our controller nomenclature) was oriented “straight ahead” relative to their body which was aligned longitudinally with the experimental room. We have modified Figure 1B and revised the Methods section to explicitly clarify this initial alignment and the instructions provided to participants.

In the revised manuscript, we have clarified that while the participant’s report is world-centered, the retinal curl provides a gaze-relative heading signal. Although this was already mentioned, we emphasize this point. In natural navigation toward a fixated target, a world-centered vector is often unnecessary; an error signal indicating heading relative to fixation is sufficient (as the reviewer also notes). However, the initial alignment of the heading within the 3D scene allows the brain to “calibrate” this internal controller, mapping the retinal curl signal onto the 3D world coordinates required for the task. Ad commented above, a new section in the discussion addresses and hopefully clarifies the relation with the controller.

The reviewer also asks how we can be certain that participants were reporting in world coordinates rather than an alternative frame, such as “heading relative to the fixation target.” We believe our “Cancelled Curl” (and over-cancelled) conditions provide the most compelling evidence to rule out this alternative. In these conditions, the physical position of the fixation target in the scene remained identical to the unaltered flow condition. If participants were simply reporting heading relative to the fixation target’s spatial location, the observed biases should have persisted regardless of the flow manipulation. Instead, the bias vanished when the curl was removed. This causal evidence proves that the bias is driven by the retinal motion signal (curl) rather than the spatial orientation of the eyes or the target’s position in the scene. Furthermore, the temporal evolution of the response supports a world-centered integration (in agreement with Warren et 2001 Nat Neuro.). For simulated straight paths, the perceived heading remains straight for the first few seconds (consistent with the initial world-centred alignment), with biases only emerging after approximately 3 seconds of integration (a point we elaborate on in our response to Reviewer #3). Had participants been responding based on a simple gaze-relative reference frame from the onset, these biases would have manifested significantly earlier. We have incorporated these points into the revised Discussion to better frame our findings alongside other cues, such as the Focus of Expansion (FOE) and egocentric visual direction that contribute to heading estimation.

Finally, we have rephrased the abstract sentence for clarity. However, we maintain that the original premise remains valid once the world-centered initial heading is aligned with the gaze-centered reference frame.

(b) It seems to me that retinal curl will depend on other variables, in addition to heading relative to the fixation target. For example, it seems to me that the magnitude of retinal curl will depend on self-motion speed, the depth structure of the scene, the angle of elevation of the fixated target, and perhaps others. This is not discussed at all, and many readers would get the misguided impression that there is a 1:1 mapping from curl to heading (relative to fixation). If I am right that this is not correct, it means that retinal curl can tell the observer whether to steer right or left to move toward the fixated target, but it cannot tell them how much to steer. Indeed, in the authors' controller model, there is a free parameter that calibrates curl to angle. It makes sense that this works to fit trajectory data that are given from a fixed environment, but it is unclear how the brain would use retinal curl to control steering when these other variables are uncertain or changing unpredictably. Moreover, how does the system change the mapping from curl to steering command as the location of fixation changes relative to the current heading? These are issues that need to be brought up in framing the problem and discussed at some length. If the authors can show mathematically that retinal curl is only dependent on heading (relative to fixation) and not any of these other variables, it would be very valuable to show the equations for this relationship.

The reviewer notes that we must be clear about the relationship between curl and heading (relative to fixation) and the variables that affect curl. We also thank the reviewer for encouraging to add the equations that show the relation of curl with additional variables. We have now included these equations in appendix 1.

Beyond the discrepancy between heading (θ) and gaze (ψ), curl is geometrically determined by translational self-motion speed (v), eye height (h), and pitch (α). More specifically, curl = (v.sinψcosα)/h. The derivation is now included in appendix 1. Since h = dsinα, where d is the 3D distance to the fixation point, we could express cos α as a function of distance. Certainly, there is not a 1:1 map from curl signal to heading relative to gaze (e.g. θ-ψ). Participant would need to know v and eye height plus extra-retinal information. Frenz et al (2003, Vis Res.) showed that people can estimate self-motion directly from optic flow, across different simulated eye height and gaze angle; extra-retinal information can, in addition, provide knowledge to ψ and α. It is then plausible that the visual system can use and transform the curl signal from a qualitative directional cue (i.e. steering left or right of fixation) into a quantitative steering command. By combining curl with knowledge of gaze orientation and eye height, the visual system can resolve ambiguities in the flow field and utilize curl as a more precise error signal for locomotor control. These aspects are now included in the new version of the discussion.

(2B) I also feel that there is a mismatch between what the behavioral task requires and what the controller model does. Subjects are apparently asked to report their heading relative to the world, but the controller model only controls their heading relative to the point that they are fixating. I understand how this is resolved in the model, but I think this type of distinction is buried and will not be apparent to most readers. Again, the reference frames of what is being measured and controlled need to be specified explicitly in all parts of the paper, and the authors need to explain how the system would combine curl-based control with some other measures of (at least initial) heading for world-centered heading to be computed. All of the assumptions need to be clearly specified.

We thank the reviewer for this point. We have addressed the alignment of the reference frames in our response to Issues 1a and 2a. Once the initial orientation (θ_0_) is established in the world frame, the controller model generates steering adjustments that directly translate into heading predictions within that same world reference frame. By treating the perceptual report as an output of the locomotor controller, we resolve the discrepancy between the steering task and the reported heading.

(2c) In addition, I found it frustrating that the authors never present raw perceptual data from the observers. Rather, in Figure 2, we see reconstructed trajectories that are perfectly smooth with no indications of noise whatsoever. Since these paths are computed from the perceptual reports, there must be some noise inherent in them. The figures should represent this uncertainty somehow, and it should be explained how these perfectly smooth trajectories are obtained.

We respectfully disagree with the reviewer’s interpretation regarding data smoothing. The thin lines in Figure 2 represent the mean 3D paths derived directly from the response variable (θ_t_) across trials of identical conditions for each participant (as detailed in the ‘Computation of Perceived Path’ section). No smoothing or filtering has been applied to these plotted trajectories other than computing the mean across trials. We also wish to remind the reviewer that the raw data and analysis code remain publicly accessible for further inspection. Having said that, we include now a supplementary figure showing an example of raw data responses as a function of time. This figure will be a supplemental figure of main Figure 2 (now provisionally included in the Suppl Information).

Regarding the visual representation: in earlier versions of the manuscript, we included shaded 95% Confidence Intervals (CIs) in Figure 2. However, this addition rendered the plot overly cluttered and obscured the individual trajectories. We therefore chose to present individual participant means (thin lines) alongside group averages (thick lines) to emphasize inter-subject variability. For clarity, the 95% CIs are explicitly displayed in Figure 3, where the data density is more conducive to shaded areas.

(3) “...the magnitude of retinal curl in the fovea can specify the body trajectory relative to gaze (Matthis et al., 2022)." The main idea put forward by the authors here seems to overlap heavily with this statement that they attribute to Matthis et al. 2022. While I think this paper still adds importantly to the topic, the authors do not discuss how their findings are different from those of Matthis et al. 2022, why they are an important extension, etc. Readers should not have to go read this other paper to have any idea how the present findings are placed in importance relative to the literature.

We have updated the Discussion to more specifically align our findings with Matthis et al. (2022). We emphasize that our study provides the perceptual validation for their ecological observation that the FOE is often too unstable for reliable use, whereas foveal curl remains a robust signal for path estimation. Our paper provides the causal link, since we manipulate curl in real-time (the ‘cancelled & over cancelled curl’ condition) providing the critical evidence that perceived heading is affected by this signal. The relation with this previous study is made clear in the revised discussion.

(4) The analysis and treatment of eye movements is extremely weak. The authors discarded trials for which gaze deviated from the fixation point by more than 3 degrees (which is a LOT given that the eye speeds are generally in the neighborhood of 0.5 deg/sec), and they provide basic stats on the distribution of positions. But this largely misses the point: it is not small position errors that are likely to matter, but rather velocity errors. Even a small amount of retinal slip of the target while it is being pursued will cause image motion that is going to alter the optic flow field around the fixation target. So, for example, the retinal curl field may no longer be centered on the fixation target. How do we know that some of the perceptual biases are not influenced by image motion resulting from imperfect tracking of the fixation target? This needs to be analyzed and discussed.

We thank the reviewer for noting that retinal slip (velocity error) is a more critical metric than positional gaze error. We agree that tracking inaccuracies can introduce translational noise into the flow field. The 3° threshold was established based on the eye tracker’s specifications and the naturalistic setup (1-meter viewing distance without head stabilization). Across all participants, the mean positional error ranged from 1.016° to 1.5° (1 deg is 2.08 cm in our setup). We also calculated retinal slip values, which ranged from 0.12 to 0.27 deg/s (X dimension) and 0.12 to 0.23 deg/s (Y dimension). These values are comparable to natural oculomotor drift (Kowler et al., 1979) and are understandably small given the low velocity of the fixation target. We have added this information about retinal sleep at the beginning of the results section.

Consequently, it is highly unlikely that retinal slip influenced the results. Furthermore, assuming that tracking error remained consistent across fixation conditions, any present retinal slip cannot explain why the bias followed the retinal curl manipulation as predicted by the controller. We therefore consider retinal slip to be an unlikely confounding factor.

(5) I found the sections of text comparing the separate and joined fits (starting line 287) to be a bit too rosy. The authors show the separate fits in the main text, and it is not very surprising that these fits are good, given that the model has 30 parameters, and these data are pretty low-dimensional. The authors only show the joined fits in the supplement, and they say that they are almost as good as the separate fits (indeed, they are better in a model comparison sense, but this is 30 parameters vs. 2 parameters). However, when I look at the fits of the joined model in the supplement, I don't find them to be very impressive. In particular, the model grossly misses the data for the straight paths for several subjects (e.g., id5, id6, id8, id10). And fitting the straight paths would presumably be easiest. This implies that the joined model is really missing something and that fitting the curved paths interacts strongly with fitting the data for different fixation target locations on the straight path. I think that the authors should discuss the results a bit more soberly and tone down their conclusions here.

We thank the reviewer for the opportunity to clarify the logic behind our modeling choices. We acknowledge that the “separate fits” are inherently less informative due to the high number of free parameters relative to the data. Our primary scientific goal was not to achieve perfect descriptive accuracy via 30 parameters, but to test a specific functional hypothesis through the “joint fit.”

The Logic of the Joint Fit:

We agree with the reviewer that the joint fit misses some paths in some conditions. Of course, the joint fit reflects a significant compromise. The “Gain” (the weighting of the curl signal) is likely not a static constant but is dynamically tuned based on task demands, confidence in the visual signal, simulated speed, and so on. By using a single Gain parameter, we intentionally ignore this contextual variability to see how much of the behavior can be explained by a “minimalist” controller. In this sense, the 2-parameter joint model is a deliberate attempt to test this limit. By forcing a single Gain parameter to account for all conditions across both straight and curved paths within one flow manipulation (e.g. unaltered flow) we are asking if a single, fixed linear relationship between retinal curl and steering effort/gain can explain the results. We view the joint fit not as a “perfect” model, but as a stronger test of the curl-based control theory. The fact that a 2-parameter model can capture the direction and scale of biases across such a diverse set of conditions (straight/curved paths, five fixation eccentricities) suggests that retinal curl is a robust signal. Upon closer analysis, these discrepancies between the joint model and the data are most pronounced in the over-cancelled condition which is the one when sensory evidence becomes more ecologically inconsistent with the extra-retinal information (gaze direction). While the joint fit successfully demonstrates that a single parameter can capture the general functional role of curl, it fails to account for the complex sensory re-weighting that occurs in ecologically inconsistent conditions (like ‘over-cancelled’ flow). We have updated the manuscript to discuss these limitations in the “fitting the controller” section, framing the model as a parsimonious first-order approximation rather than a complete description of human heading perception based on a minimal set of parameters.

(6) The section of the paper on neural simulations (starting line 387) has a few weaknesses. First, why are only straight paths simulated here? This does not seem to provide a very rigorous test of the model. Second, it is awkward that the simulation results are presented in units of pixels, rather than degrees. Third, the authors seem to downplay the fact that the neural estimates of heading seem to oscillate rather wildly (over a range of hundreds of pixels, whatever that means, see especially Figure S16). It was far from clear to me how an estimate of heading with these large oscillations is useful. It would seem to require that heading estimates are integrated over substantial lengths of time to be reliable. It was therefore unclear how the model produces such smooth paths from these oscillating estimates.

We acknowledge that the presentation of the neural model requires more clarity regarding its objectives and its relationship to the behavioral data.

We first wish to clarify the intended scope of the neural ring-attractor model. Our primary goal was not to provide a comprehensive account of behavioral performance across all conditions (which is the role of the controller model), but rather to demonstrate a biologically plausible mechanism that explains the emergence of the “Opposite-to-Gaze” bias. While the controller demonstrates that the bias follows a specific control law, the neural model shows how such a law can emerge from known primate neurophysiology, specifically, spiral-tuned MSTd neurons, gaze-contingent inhibition, and an egocentric “straight-ahead” prior.

Why Straight Paths are Sufficient for this Objective. The reviewer asks why only straight paths were simulated. In our study, the straight-path condition with eccentric gaze is the purest test of the bias mechanism. Simulating the straight paths allowed us to isolate the interaction between foveal inhibition and the straight-ahead prior without the confounding variable of path-curvature flow. Given the complexity of the neural network’s parameter space, we focused on these conditions to provide a clear neuro-plausible explanation. We have added text when introducing the model (Neural simulations in the Results section) to make clear why we model straight paths only.

Units: Pixels vs. Degrees. We acknowledge that the use of “pixels” in the plots of internal neural dynamics may appear awkward. The neural network operates on input stimuli that are defined by the pixel resolution of the videos used in the simulations, we used pixels as the native coordinate system to describe the movement of activity peaks within the network’s internal “map.” We have decided to keep the pixel units in these figures.

Behavioral Output (Meters): Importantly, the final heading estimates produced by the network are not left in pixels. We use a pinhole camera model to reconstruct the 3D trajectories from the neural activity. These results are expressed in meters, allowing for a direct comparison with the human behavioral data.

Addressing Wild Oscillations and Smooth Paths. The oscillations observed in the instantaneous heading estimates reflect the stochastic nature of the population peak when tracking high-frequency sensory inputs. In our model, the synaptic time constant (τ) was kept relatively small to ensure a fast, low-latency response to changes in self-motion. While increasing τ would have produced smoother internal dynamics, it would also have introduced delays into the control loop. Instead, we chose to maintain this high sensory responsiveness and applied a temporal moving average later to the network’s decoding to reconstruct the 3D trajectories. This is explicitly stated in the section “Heading Estimation and 3D path reconstruction” in the new appendix 2.

In addition, the neural activity over time is shown in two ways: the heatmap shows the neuron with preferred heading one can see more oscillations, specially when the fixation point is closer to the centre (eccentricities -2 and 2), due to larger competition between the sensory evidence and the straight-ahead prior. The other way is the decoded heading. In the ring-attractor model, the decoded heading (φ̂) is not determined by a single neuron but is calculated using a population vector average (equation 19). By summing across the entire population, the decoder effectively integrates sensory evidence from many neurons simultaneously. One can appreciate (see e.g. Fig. 5B) that averaged decoding, leads to a smoother resulting estimate (the white dashed line, whose visibility had been improved in the revised version). Behavioral work by Burr and Santoro (2001) suggests that global motion signals (divergence and rotation in optic flow) are integrated over much longer timescales—roughly 1000ms to 3000ms—compared to local motion units (~200 ms).

In the previous manuscript, we discussed this aspect in lines 424-426. In the new version, we have added text in the Heading estimation and 3D path reconstruction section (now in appendix 2) stating that we smoothed the decoded signal in agreement with this psychophysical evidence before applying the camera model.

See also our comment on temporal integration in the responses to reviewer #3

**Reviewer #2 (Recommendations for the authors):**

(7) Line 51: "...a functional role of rotational flow components has been largely neglected in both theoretical and experimental work on heading perception." I feel like this statement is too strong and that the authors try too hard to "sell" their findings by underrepresenting previous work. There are numerous studies (many not cited), both behavioral and electrophysiological, that have examined how heading perception depends on pursuit eye movements, either physical movements or visually simulated ones. These studies directly involve rotational flow components, and several of them have concluded that rotational flow components contribute to estimating heading in the presence of eye movements (just one example is Grigo and Lappe 1999). Because these studies generally involved horizontal pursuit of a target on the horizon, rather than tracking a point in the ground plane (like the authors' work), these studies generally did not involve flow fields with retinal curl around the fixation point. But I consider these older studies just a special case of the more general geometry, and they still involve rotational flow components. Moreover, various previous studies have used stimuli for which there was no FOE present in the visible display (either due to simulated rotation or masking out the FOE), and the authors do not seem to give credit to these works either. In addition, several studies have implicated a role of extraretinal signals in perceiving heading during eye movements, so retinal curl cannot explain everything. Rather than overemphasizing the limitations of previous work, the authors would be better served to explain how their findings extend and generalize from these previous studies.

We thank the reviewer for pointing out this oversight; it was not our intention to overlook previous work. While our original version cited studies considering rotation-related cue, we have now substantially revised the introduction to include previous work and better acknowledge the informative role of rotation. Our central aim remains to distinguish between models that compensate for rotation to recover a heading vector and our proposal that the visual system exploits retinal curl directly as a primary, functional signal for locomotor control.

We have now updated the Introduction and Discussion to better situate our work within the context of studies (including Grigo & Lappe, 1999 and some additional ones we have included in the new version) that have investigated the informative role of rotational flow. We now clarify that our study extends these findings by investigating the non-uniform rotational patterns (curl) that emerge during ground-plane fixation, representing a more general and biologically ubiquitous case of locomotor control, while acknowledging previous studies that have also considered the potential role of curl generated by gaze fixation.

(8) Figure 3: I did not understand why there are two purple and two blue curves in the graphs of the middle column. And the caption does not explain this.

This a very good observation. This was explained in lines 268-273 (previous version). When the gaze is straight-ahead (same direction as heading), there is no curl. However, we introduced positive or negative curl in the altered conditions. These purple and blue lines refer to these trials and show that the bias re-appears in the expected direction when curl is (unexpectedly) added. We think this adds additional evidence to the curl contributing to heading. Even though this was extensively explained we have added text in the caption of figure 3.

(9) Line 331: What makes the authors think that retinal curl is computed in area MSTd? They should cite studies to support this idea if it has been shown in physiology.

Evidence was cited in the introduction (Graziano et al 1994) of sensitivity to spiral motion in addition to neuro-computational models that implement this activity also cited (e.g. work of Leyton et al.)

(10) The neural network model for computing heading from curl requires a "gaze-centered inhibitory drive" that inhibits activity around where the eyes are looking. This is probably a biologically plausible thing, but is there any evidence to support the idea that this signal exists in the parts of the brain where the authors believe these computations to be happening? They simply posit the existence of this gaze-centered inhibition as though it is common knowledge, but they provide no citations nor discuss any previous evidence for its existence.

While neurophysiological evidence primarily describes this as gain-field modulation, this process frequently involves localized suppression of neural activity to facilitate coordinate transformations. In parietal areas such as LIP and 7a, eye-position signals do not just enhance responses but can also suppress them, effectively shifting the 'center of gravity' of a population response (Read et al 1997; Born et al 2005, cited in the discussion in the revised section re-evaluating the Focus of Expansion). In the context of our ring-attractor model, this functional modulation is most parsimoniously implemented as a gaze-centered inhibitory drive.

(11) Lines 482-483: Why should perceptual biases related to retinal curl take seconds to show up?? The curl information itself must be present very quickly, perhaps requiring just a few video frames. So what does this imply about mechanisms? The authors throw out this assertion, but it is left hanging without any further analysis or support.

The time course reflects the integration requirements of complex motion processing. While local flow is processed rapidly, global patterns like retinal curl require longer temporal windows to reach a stable estimate (Burr et al 2001). In our study, this integration is functionally necessary to filter the higher-frequency 'wobble' induced by gait-cycle oscillations. We now discuss the temporal integration aspects under a new heading in the discussion.

(12) Line 508: "This suggests that the "bias" observed in our perceived headings may reflect the operation of a control law optimized for action rather than a failure of a perceptual system designed for passive estimation." The authors make this statement to justify why perceptual biases are present with unaltered curl. But I don't fully understand the logic. Are they saying that it is not possible to have a set of computations that can do both things accurately? Is it possible to show this theoretically? Moreover, if it is not possible to rule out other possible sources of the biases, such as those described above (reference frame of judgments, eye movements, etc), then is it necessary to invoke this logic?

Our logic is that the observed 'bias' is not a representational failure, but a functional byproduct of a control law optimized for active steering. In a closed-loop system, the objective is to null the error signal (retinal curl) to maintain a stable path. When observers are asked to make an open-loop heading report, they likely utilize this same control signal, which manifests as a systematic bias toward the 'null' point of the controller as a result of a sustained fixation in discrepancy with the simulated translation/heading.

We do not suggest that accurate perception and control are theoretically incompatible; rather, we suggest that perception and action rely in the same underlying information (e.g. work of Brenner & Smeets). While other factors, such as coordinate transformations between reference frames, certainly can contribute to the reporting process, our interpretation provides a parsimonious link between the psychophysical data and the underlying steering mechanism. By framing the bias as a consequence of a 'nulling' strategy, we explain not just the existence of the error, but its specific direction and magnitude relative to the fixated target.

(13) Line 546: "...MSTd would simultaneously code curvature for trajectory estimation and heading across the neural population, with curvature encoded through the spirality of the most active cell and heading through the visuotopic location of its receptive field center." The latter part of this argument seems to imply a relationship between the heading preferences of MSTd neurons and the locations of their receptive fields. I am not aware of any evidence for such a relationship, so the authors should indicate whether this is based on some experimental data or just a speculation.

We thank the reviewer for this observation. The proposal that heading is signaled by the visuotopic location of active MSTd populations is a core architectural feature of our model and is supported by several lines of evidence. In the Layton and Browning (2014) framework, MSTd is modeled as a visuotopic map of functional 'hypercolumns'. Each hypercolumn contains neurons tuned to a continuum of spiral patterns, but all neurons in a given hypercolumn share a receptive field center at a specific location in visual space. Consequently, the visuotopic location ($x, y$ coordinates) of the maximally active hypercolumn represents the center of motion (heading), while the spirality (the tuning dimension within that hypercolumn) represents path curvature. We have clarified this in the revised discussion (re-evaluating the FoE) to emphasize that this dual-coding scheme arises from the simultaneous representation of 'where' (population map location) and 'what' (spiral tuning) in MSTd.

**Reviewer #3 (Public review):**

The primary limitation of the paper is that it avoids discussion of some of the inevitable complexities of heading perception. The main issue is what exactly is meant by heading. Different behaviors evolve over different timescales. The geometry of retinal motion defines instantaneous heading, which varies widely through the gait cycle. Time-varying information like this is known to be important in the momentary control of balance. Heading can also be thought of as steering the body toward a distant goal, which evolves over longer timescales. The current manuscript appears to be concerned with heading information integrated over a few seconds and seems to provide evidence that heading is indeed integrated over the gait cycle. The issue of the time scale of the computation is touched on, but it is not related to how it might be used in normal walking or what situations it might apply to. Steering toward a distant goal during walking is not a very difficult problem and may not require evaluation of retinal motion, but control of balance is more challenging and may depend critically on curl. Consequently, the timescale of the computation needs to be considered in order to understand what is meant by heading.

We thank Reviewer #3 the comments regarding the definition of heading at different time scales, the role of the gait cycle, and the temporal integration of the curl signal. These comments have helped us refine the manuscript’s core arguments.

We agree that “heading” must be precisely defined within the context of the differing temporal demands of balance and steering. While instantaneous heading provides the high-frequency feedback necessary for momentary postural adjustments and balance, our study is concerned with heading as a gaze-relative signal used for the continuous control of a locomotor trajectory. As such, we have revised the manuscript to specify that the perceived heading measured in our task reflects a signal integrated over the gait cycle to filter out the oscillatory noise induced by head bob and sway (mainly in the Discussion section).

The reviewer correctly notes that gait-induced head bob and sway produce high-frequency oscillations in the curl signal, yet our behavioral results show smooth, slowly evolving biases. The visual system does not react to “instantaneous” curl, which would lead to jittery, unstable heading estimates. Instead, it integrates flow over a timescale roughly commensurate with a full gait cycle (~500–1000ms). This implies a significant temporal integration process. This temporal integration is consistent with evidence (Burr and Santoro,2001, Vis Res) indicating that optic flow signals (radial and rotational components) are integrated over windows of approximately up to 3 seconds to ensure perceptual stability. Neurally, this likely involves the projection from area MSTd to the Ventral Intraparietal area (VIP), a pathway where fast, eye-centered sensory inputs are transformed into stable, body-centered representations suitable for guiding long-term steering behavior (Chen et al. 2011, JNeurosci.). By grounding our definition of heading in these specific temporal and neural constraints, we tried to clarify how the visual system exploits retinal curl for goal-directed action in natural, dynamic environments and relate our findings to recent studies addressing the role of retinal motion on balance (Powell et al. 2026 Bioarx).

In our implementation, we explicitly address the high-frequency noise introduced by gait dynamics by smoothing the retinal curl signals computed from the stimulus videos before they are fed into the controller. This temporal filtering allows the fit of the controller’s prediction to the response data while remaining robust to the rapid fluctuations of head bob and sway. In contrast, the neural ring-attractor model would not require an external smoothing step; instead, the integration is an emergent property of the system’s architecture that can be controlled with different parameters, as commented above in a response to Reviewer #2. The dynamics of the synaptic weights and the characteristic “leak” in the population activity naturally implement a leaky integration of sensory evidence, ensuring that the decoded heading reflects a sustained estimate rather than an instantaneous response to visual noise.

We also agree that we avoided discussing some complexities of the heading perception. In the new version, we also include and integrate the distinction between instant heading and future path in different parts of the ms (introduction) and mainly discussion (temporal integration and steering section) which have been revised substantially.

**Reviewer #3 (Recommendations for the authors):**

There are a number of points that require clarification.

(1) Head bob and sway were included in the stimulus and need to be addressed in both the analysis of the data and the interpretation. The curl signal in the stimulus varied over time, commensurate with normal gait. However, the results don't reflect the same level of variability that would be produced from curl over the gait cycle. This means that the information must be integrated over some longer timescale. It is not clear from the data analysis what this integration is. Is there an implicit integration with the manipulation of the steering wheel? If subjects indeed appear to be able to use curl to evaluate heading over timescales of seconds, this needs to be explicitly addressed, as it is a novel result. This would require parts of the discussion to be changed/expanded to maintain consistency. For example, line 482 talks about the buildup of biases over time.

As commented above in the public response, the curl estimated from the optic flow algorithm was smoothed before being input into the controller (path fitting and predictions). The smoothing was only applied to the curl signal, not to the participants responses. Also, as mentioned before, the time course of the bias is consistent with integration times of optic flow reported in the literature. All these aspects are now explicitly included in the new display and conditions section (Flow manipulation conditions).

(2) Since the experiment included curl variability resulting from the gait cycle, some discussion is needed about the role of retinal motion in the control of balance and posture. There is a large literature about the role of flow in controlling gait and momentary adjustments of the body while walking. Additionally, it should be noted that in the task, head bob and sway from 1 prerecorded individual was shown to all subjects. It is known that gait varies significantly between different individuals, and it should be acknowledged that this may lead to differences at the individual subject level for perceiving heading.

We have included a point in the discussion addressing the different time scales for different use of optic flow signals (postural control vs locomotion).

We agree with the reviewer that utilizing a single gait profile for all participants may introduce individual differences in perceived heading, as the simulated head motion might not perfectly match each participant’s unique biological gait signature. However, we prioritized stimulus consistency over idiosyncratic accuracy. By ensuring that every participant viewed the exact same motion profile, we could be certain that the systematic 'opposite-gaze' biases observed across the population were driven by our experimental manipulations of gaze and retinal curl, rather than being confounded by variability in head-motion kinematics. We have added an acknowledgement of this point at the first paragraph of the displays and conditions section in the Methods.

(3) More information is required about the use of the rotating wheel for the measurement of heading. How easy was it to use? What about time delay, and how does this deal with the bob and sway? Does the wheel impose a de facto integration on the perceptual measurement?

The rotary encoder provided an intuitive steering-wheel interface that participants found easy to operate. To ensure minimal latency (1–5 ms), the device was interfaced via an Arduino Uno and sampled by a dedicated background Python thread, isolated from the visual rendering loop. We have incorporated these technical details into the Methods (Procedure) section.

(4) Restructuring the description of the models It remains unclear why the dynamics of the neural network are a necessary inclusion in this paper. It seems interesting, but there is no comparison to actual neural data or other related work. Instead, this appears to be a description of what the network is doing, which is already defined by the equations. This needs to be clarified for its exact interpretation with respect to real neural data, and its importance here for understanding the biases that emerge in heading judgments. The paper would flow better if this section were included as supplementary material or omitted from the paper entirely, as it seems to detract from the other points. If this is a description of why the biases are seen in the controller, then the supplementary material is a good place for it.

We thank the reviewer for this suggestion. We clarify that the neural model is not intended to simulate specific empirical neural data, but rather to provide a biologically plausible implementation of the controller. This allows us to demonstrate how the observed biases emerge from the dynamics of standard cortical architectures, such as ring attractors. This is now mentioned when introducing the neural simulation results.

Following the reviewer's suggestion, we have moved the neural model equations to Appendix 2 while retaining the simulation results in the main text (Results). We believe it is essential to present not just the abstract controller, but also its functional implementation, as this provides a mechanistic bridge between retinal signals and locomotor behavior.

(5) For the modeling approaches, the math would be more appropriate for supplementary materials.

To ensure a better flow of the paper, we have moved the neural model equations to Appendix 2, while Appendix 1 now details the relationship between measured curl and other variables (speed, yaw, pitch, etc.). We have retained the controller model in the Methods section, consistent with the eLife layout where Methods follows the Discussion.

(6) How do the models and data analysis deal with the influence of the gait cycle in the input? Do they integrate the information over that timescale? If so, the integration time needs to be specified.

As commented above, to mitigate gait-cycle fluctuations, we smoothed the computed curl signal before inputting it into the controller and applied a similar smoothing process to the neural model’s readout. Using a LOESS filter, the effective integration window was 2.4 seconds. Close to the integration time reported in Burr et al. 2001. These parameters have now been explicitly specified in the Methods section. For the empirical data, we just utilized trial-averaging.

(7) What does biologically plausible mean in terms of the neural network model? Especially when control wasn't explicitly a variable in the measured behavior of the subjects.

By biologically plausible, we mean that our model is constrained by neural architectures documented in the primate brain—specifically ring-attractor dynamics, population coding, and gaze-centered gain-fields. Crucially, the network utilizes recurrent connectivity with a 'Mexican-hat' profile (local excitation combined with lateral inhibition). This is a standard and widely accepted motif in computational neuroscience, representing the consensus on how cortical circuits maintain a stable "bump" of activity to represent spatial variables. Rather than introducing ad-hoc mechanisms, we demonstrate that the observed behavioral biases emerge naturally from these established neural components when they are tasked with maintaining locomotor stability. Since we think this aspect was already emphasized, we haven’t added any additional detail.

(8) There should be more extensive acknowledgement of the body of literature that has challenged the use of the focus of expansion. That section should also include references to work that has investigated extraretinal signals, as they may also be important.

We have expanded the Introduction and Discussion to more thoroughly acknowledge research challenging FOE-based models and the critical role of extraretinal signals. These updates, which also align with our response to Reviewer #2, provide a more comprehensive context for our model within the existing body of heading and self-motion literature.

Minor points:

(1) Line 69 - In self-generated motion, spiral patterns are almost always centered on the fovea, but many physiological experiments present spirals in the peripheral retina. This is incompatible with the motion generated during self-motion. Therefore, clarify whether the type of spiral motion Graziano investigated was centered on the fovea.

In the experiments conducted by Graziano et al. (1994), spiral stimuli were centered on the receptive field (RF) of the individual neuron being recorded to accurately characterize its tuning. While the reviewer correctly notes that spiral centers often align with the fovea during active steering (due to fixation on a goal), MSTd neurons possess large RFs that provide a comprehensive 'template' system across the visual field. This population-level representation allows the brain to recover trajectory information even when the focus of motion shifts relative to the fovea—for example, during pursuit eye movements or when fixating on landmarks off the direct path of travel. To keep this part of the text brief, we haven’t add more details concerning this study.

(2) Line 72 - "Magnitude" instead of "amount".

This has been changed.

(3) Line 115 - State explicitly whether the scale of the visual stimulus was matched to the scale of the actual natural images shown in VR.

We have updated the Methods (Displays and conditions) section to explicitly state that the visual scale was veridical. The virtual camera’s parameters were calibrated such that its field of view (91°) matched the physical dimensions of the projection screen (2.03 m × 1.16 m) at the 1.0 m viewing distance. This ensures that the angular size of the objects and motion gradients in the stimulus were 1:1 with the scale of the simulated natural environment.

(4) Line 144 - While Farneback is a good dense flow estimation algorithm, it is noisy and may impose biases/variability in the calculation of curl. This should be acknowledged.

We acknowledge that the Farneback algorithm can introduce variability in curl estimation. To mitigate this, we utilized 10 independent renderings of each experimental trial to compute the flow fields. Although this methodology was reflected in the data previously uploaded to our OSF repository, we have now explicitly added this detail to the manuscript (Flow (curl) manipulation conditions). The computed curl used for the modeling was derived from the aggregate of these different runs, ensuring a robust and stable signal that accounts for potential algorithmic noise.

(5) Line 206 - "Join fits". Is this a technical term? It sounds awkward. Would "Joint fits" make more sense?

The referee is right. We have corrected this.

(6) Line 280 - "Consistent with" (typo).

This has been corrected.

(7) Line 286 - Typo in title.

Also corrected to Fitting the controller.

(8) Lines 482-493 - There should be more discussion on the time course of integrating the stimulus, and the relationship/generalizability to more natural stimuli.

This part of the discussion (related to integration time) has been changed considerably to include discussion of postural control in addition to locomotion.

(9) Lines 516-517 - It is mentioned that retinal flow dynamics override the visual direction cue. This may not be generally true, as the reweighting of the cues might depend on things like task demands or actual stimulus context. In the present experiment, the subject only has access to a large moving textured ground plane, and the body is stationary.

We agree with the reviewer that cue reweighting is highly context-dependent. However, as noted in the original manuscript (Lines 516-517), we specifically stated that retinal flow dynamics 'can' override the visual direction cue, rather than asserting a universal rule. This phrasing was intentional to acknowledge that while flow is a potent signal—especially in the presence of a large, textured ground plane as used in our paradigm—the relative weighting of these cues remains contingent on the specific sensory and task conditions. We believe this remains a fair and cautious interpretation of our findings.

(10) Lines 524-256 - It is unclear why Matthis et. al. 2022 is cited for this point. Some of the steering literature, like Wilkie Wann & Allison 2006 or Lappi & Mole 2018 (and some of their other work), would be more relevant for the definition of a control law under these circumstances.

We agree and this part has been changed substantially.

(11) Lines 528-532 - Warren et. al. 2001 should be cited in this section because their results were interpreted in terms of focus of expansion, but may result from the curl signal (Powell et. al., 2026. The Role of Retinal Flow in Walking. bioRxiv, 2026-02.).

We agree with this suggestion and the citation has been added.

(12) Lines 544-546 - Layton and Browning are focused more on path perception from the implemented spiral tuned cells. Because of this, it wouldn't be appropriate to say it is a shift away from FOE-based heading based on this citation alone. There are more models/psychophysical results that would strengthen this claim.

We agree with the reviewer that Layton and Browning focus specifically on path perception. Our original intention in citing this work was to emphasize the neurophysiological continuum from radial to circular motion (spiral tuning) rather than discrete expansion/rotation channels. However, we have revised this section to clarify that the reliance on retinal curl represents a mechanism for determining the future path (locomotor trajectory) rather than merely instantaneous heading. This distinction acknowledges that while heading is a momentary vector, the integration of curl signals allows the system to anticipate and control the intended path over time—a framing that better aligns with both the cited literature and our proposed controller model. As commented above this is now extensively discussed in the revised version.

(13) Figures - There are minor visibility issues for some of the figures. In Figure 2, the thick line is unreadable, and in Figure 1, the x-axis labels are crowded.

The axis in Fig 1 has been modified to avoid crowdedness. In Fig. 2, the thick (average line) has been modified. We hope they are more visible now.
